# Investigation of electron-induced cross-linking of self-assembled monolayers by scanning tunneling microscopy

**DOI:** 10.3762/bjnano.13.39

**Published:** 2022-05-25

**Authors:** Patrick Stohmann, Sascha Koch, Yang Yang, Christopher David Kaiser, Julian Ehrens, Jürgen Schnack, Niklas Biere, Dario Anselmetti, Armin Gölzhäuser, Xianghui Zhang

**Affiliations:** 1 Physics of Supramolecular Systems and Surfaces, Faculty of Physics, Bielefeld University, 33615 Bielefeld, Germany; 2 Department of Chemical Engineering, Imperial College London, London SW7 2AZ, United Kingdom; 3 Condensed Matter Theory Group, Faculty of Physics, Bielefeld University, 33615 Bielefeld, Germany; 4 Experimental Biophysics and Applied Nanoscience, Faculty of Physics, Bielefeld University, 33615 Bielefeld, Germany

**Keywords:** carbon nanomembranes, electron-induced cross-linking, scanning tunneling microscopy, self-assembled monolayers, subnanometer pores

## Abstract

Ultrathin membranes with subnanometer pores enabling molecular size-selective separation were generated on surfaces via electron-induced cross-linking of self-assembled monolayers (SAMs). The evolution of *p*-terphenylthiol (TPT) SAMs on Au(111) surfaces into cross-linked monolayers was observed with a scanning tunneling microscope. As the irradiation dose was increased, the cross-linked regions continued to grow and a large number of subnanometer voids appeared. Their equivalent diameter is 0.5 ± 0.2 nm and the areal density is ≈1.7 × 10^17^ m^−2^. Supported by classical molecular dynamics simulations, we propose that these voids may correspond to free volumes inside a cross-linked monolayer.

## Introduction

Electron-induced chemistry plays an essential role in science and technology. A highly focused electron beam is employed to create nanostructures via electron-beam lithography [1], and has been further developed to produce three-dimensional structures through controlled dissociation of precursor molecules [2]. Electron-induced chemistry has also been used to explain the synthesis of complex molecular species in the interstellar medium [3]. Electron–molecule collisions have been intensively studied in the gas phase and on surfaces. Depending on the electron energy and the molecular structure, several processes may occur, such as elastic collision, rotational or vibrational transitions, electron attachment, electronic excitation, and ionization [4–5]. Oriented molecular layers on surfaces are particularly well suited for such studies as surface analytical tools, such as scanning tunneling microscopy (STM), allow for detailed observations of molecular dissociation and bond formation. In related efforts, on surface polymerization, a reaction of monomers in a two-dimensional confined space has developed rapidly in the last decades, representing a new strategy to create functional molecular nanostructures in a controlled fashion [6–9].

Self-assembled monolayers (SAMs) are ordered molecular assemblies formed by the adsorption of amphiphilic organic molecules on a solid surface [10–12]. Self-assembled monolayers are model systems that enable a fundamental understanding of self-organization and provide a versatile path to tailor surface properties [13–20]. These monolayers can be modified with lithographic tools, such as scanning probes [21], UV light, X-rays, ions, or electron beams [22–24]. A particularly versatile nanofabrication scheme utilizes electron irradiation of aromatic SAMs to create ultrathin carbon nanomembranes (CNMs) [25–26]. Depending on the precursor molecules and the exposure conditions, thickness [27], mechanical stiffness [28], and electronic transport characteristics [29–30] of CNMs can be tailored. Carbon nanomembranes have been applied as electron microscopy supports [31–32], mechanical resonators [33], and dielectrics in nanocapacitors [34–35]. It has been shown that CNMs possess subnanometer pores, which can block the passage of most molecules and ions, but let water and helium pass through, enabling the use of CNMs in molecular filtration and ion separation [36–42].

The interactions between aromatic SAMs and electrons have been studied by X-ray photoelectron spectroscopy (XPS), near-edge X-ray-absorption fine-structure (NEXAFS) technique [43–49], infrared spectroscopy [43,50–52], high-resolution electron energy loss spectroscopy (HREELS) [53–54], Raman spectroscopy [55], and low-energy electron microscopy (LEEM) [56] as well as by theoretical analysis [57–59]. It is now well established that electron irradiation leads to cleavage of C–H and S–H bonds, followed by the formation of C–C bonds between neighboring aromatic molecules. The mechanisms of cross-linking are dependent on the electron energy: below the ionization potential, a two-step resonant dissociative electron attachment (DEA) has been proposed [5,60–62]. Based on HREELS data, Amiaud et al. proposed a resonant electron attachment process at ≈6 eV and suggested that the cross-linking of aromatic SAMs proceeds in a radical chain reaction [53]. Above the ionization potential, a direct electron impact ionization is believed to cause C–H cleavage and generate radicals [63]. Neumann et al. found a strong energy dependence for the effective cross-sections of a complete cross-linking of 4'-nitro-1,1'-biphenyl-4-thiol SAMs [56]. These studies provided valuable insights into the chemical transformations associated with cross-linking. However, direct imaging on the atomic scale to visualize the evolution of irradiation-induced structural changes is still lacking. In this work, we investigated the structural changes occurring upon irradiation of SAMs of *p*-terphenylthiol (TPT) on Au(111) using a combination of scanning electron microscopy (SEM) and scanning tunneling microscopy in ultrahigh vacuum (UHV) at room temperature. To study the initial stage of cross-linking, the SAMs are exposed to 50 and 1 keV electrons at very low doses and subsequently imaged by STM. The electron dose is then gradually increased up to doses of 25 mC/cm^2^ and the electron-induced structural changes were then determined.

## Results and Discussion

**Figure 1:**
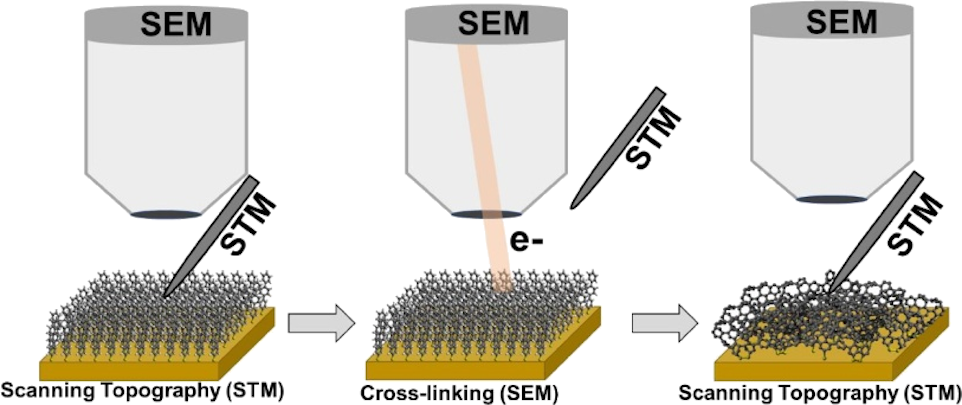
Schematic illustration of imaging pristine and irradiated self-assembled monolayers (SAMs) on Au(111) by STM. First, STM data of the pristine SAM is acquired. Second, the STM probe tip is retracted and moved to the side to enable subsequent irradiation by the SEM beam. Finally, the STM probe tip is positioned back to the initial sample location and the data of the irradiated STM area is acquired.

The general concept of self-assembly allows for the preparation of SAMs from the liquid or gas phase. Highly ordered TPT SAMs spontaneously form on Au(111) due to the formation of bonds between sulfur and gold atoms, which is accompanied by van der Waals interactions between the aromatic rings. The TPT SAMs were first imaged by STM, then exposed to 1 keV or 50 eV electrons at a series of doses, and imaged again by STM (see Figure 1).

### Characterization of TPT SAMs on Au(111)

**Figure 2:**
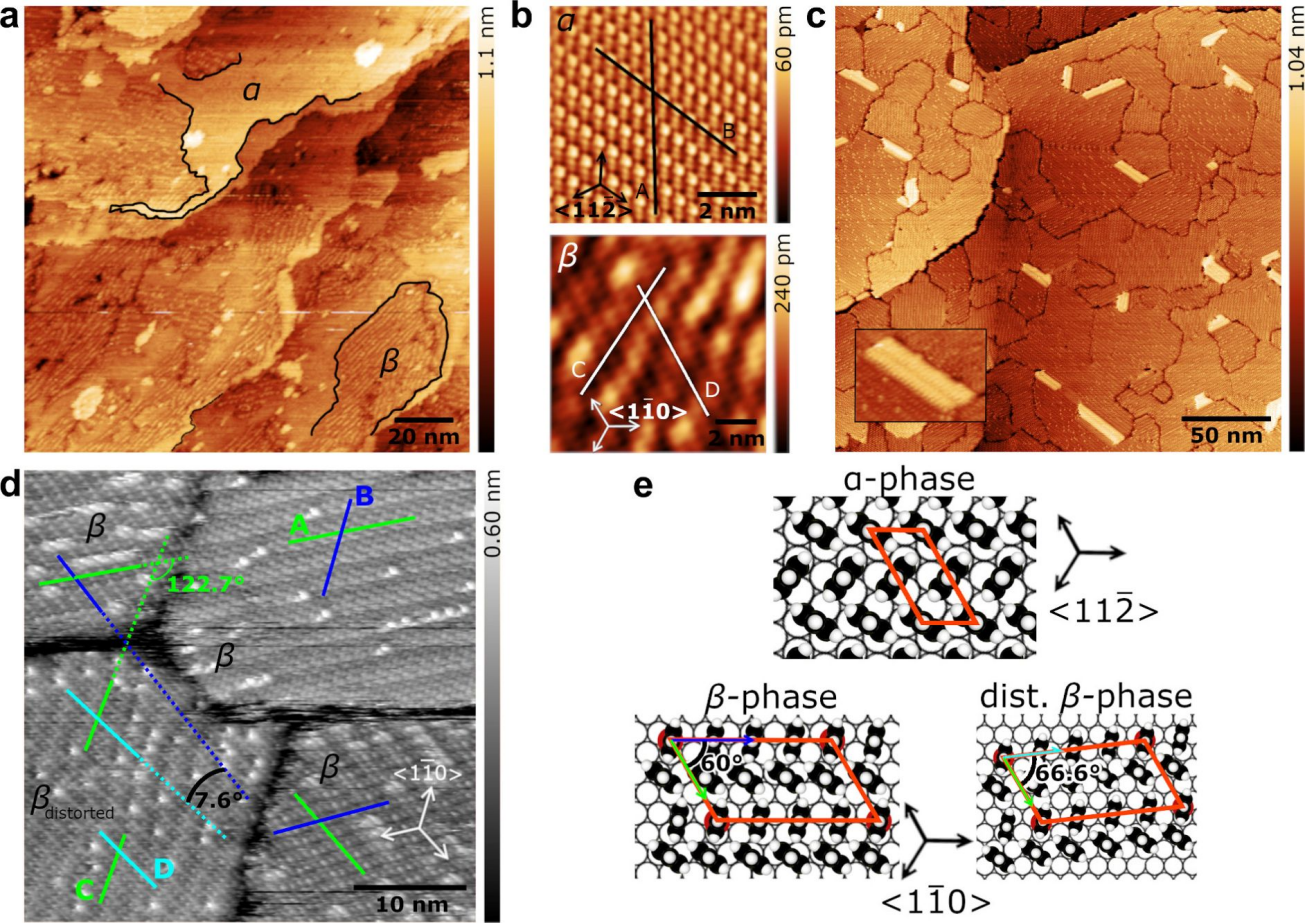
STM imaging and analysis of TPT SAMs on Au(111). (a) Low-magnification STM image (+0.4 V, 70 pA) of a monolayer prepared from DMF-based solution showing α-phases and β-phases as well as gold monolayer islands. Domain boundaries are marked by black solid lines. (b) Top panel: high-resolution image of the α-phase (+0.4 V, 100 pA); bottom panel: high-resolution image of the β-phase (+0.4 V, 70 pA). (c) Low-magnification STM image (+1.0 V, 30 pA) of monolayers prepared from the gas phase showing ordered domains with sizes of 10–100 nm. The inset shows one Au adatom island (covered by TPT molecules) with a height of 0.29 ± 0.07 nm. (d) High-magnification image (+1.2 V, 30 pA) of Figure 2c showing the β-phase and the β_distorted_-phase. The stripe directions are highlighted in green. The pair stacking direction is marked in blue for the β-phase and turquoise for the β_distorted_-phase, respectively, with a measured angle of 7.6 ± 5.0°. (e) Models for α-phase, β-phase, and β_distorted_-phase of TPT SAM on Au(111), where equivalent binding sites are highlighted.

The TPT SAMs prepared from dimethylformamide (DMF)-based solution display two molecular arrangements: the α-phase and the β-phase (Figure 2a), similarly to what have been reported [64–67]. Figure 2b (top panel) shows a fast Fourier transform (FFT)-enhanced image of the α-phase, where the absence of Moiré-like superstructures indicates a commensurate molecular arrangement with respect to the Au surface. Figure 2e (top panel) shows the molecular structure model of the α-phase, where the sulfur atoms are located on a (√3 × √3)*R*30° lattice and the molecular backbones exhibit a (2√3 × √3)*R*30° structure. The area per molecule of the α-phase is 0.216 ± 0.036 nm^2^. The tilt angle for the TPT backbone in the α-phase is ≈13° relative to the surface normal [65]. Figure 2b (bottom panel) shows a high-resolution image of the β-phase, which is characterized by rows of oval spots aligned at an angle of ≈60° with respect to the stripe direction. Each oval spot contains two TPT molecules. Such superstructure is caused by point-on-line incommensurability between the monolayer and the Au surface. The stripe direction (line C) and the pair stacking direction (line D) are two different symmetry directions. The height profiles of lines A–D in Figure 2b were shown in Supporting Information File 1, Figure S1. The unit cell is described in matrix notation as




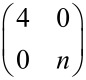




relative to the substrate crystallographic directions 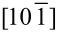 and 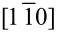, with *n* being close to 8 [65]. The area per molecule of the β-phase is 0.288 nm^2^ (*n* = 8). The corresponding tilt angle of the molecular backbone is in the range of 33–49° with respect to the surface normal. The α-phase is thus elevated by ≈0.6–0.8 Å compared to the surrounding β-phase domains.

To our surprise, TPT SAMs prepared from the gas phase exhibit only β-phase domains with sizes of 10–100 nm (Figure 2c). Notice that the sample was further annealed at 343 K for 15 min in order to remove physisorbed molecules, which results in a less dense but more ordered structure over a large area with very few defects. During the self-assembly process, gold adatoms are ejected from the surface layer due to the relaxation of the herringbone reconstruction [68]. Several gold adatom islands, which would build up if the density of adatoms exceeds that for critical island nucleation, were observed here. We also found bright features with an apparent height of 1.6 ± 0.2 Å, which does not correspond to the height (≈2.5 Å) of a Au(111) single atomic step. The β-phase is characterized by a mesh of domain boundaries with an apparent depth of 1.5 Å. A further inspection of the β-phase in Figure 2d reveals two kinds of structures: (1) The pair stacking directions (blue) of the β-phase domains are only multiples of 120° and (2) the pair stacking direction (turquoise) of the β_distorted_-phase (lower left corner) is twisted by an angle of 7.6 ± 5.0° with respect to the β-phase domains. The appearance of the oval spots depends on the relative orientation between the fast scan direction and the domain orientation. Depending on the relative orientation, the oval spots divide into two spherical spots [64]. The structural models for the β-phase and the β_distorted_-phase are shown in Figure 2e (bottom panel).

### Initial stage of cross-linking and observation of dark spots

**Figure 3:**
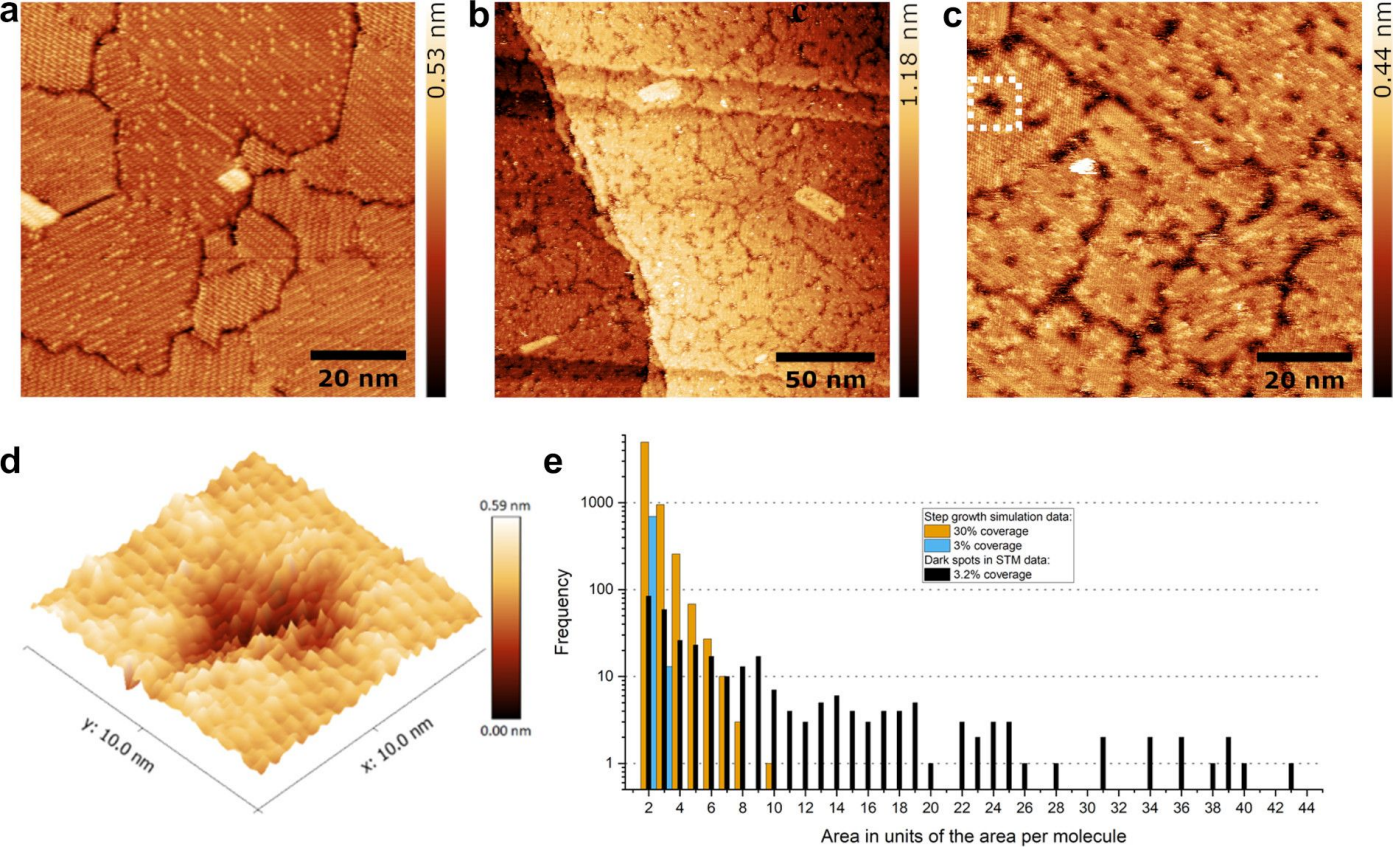
Initial stage of cross-linking of TPT SAMs. (a) STM image (1.0 V, 30 pA) of a pristine TPT monolayer prepared from the gas phase. (b) STM image (0.45 V, 70 pA) of the same sample that has been irradiated with 1 keV electrons at a dose of 0.5 mC/cm^2^. (c) High-magnification image of Figure 3b showing the emergence of dark spots. (d) 3D view of one selected dark spot (marked with a white dashed square in Figure 3c) of the monolayer, where molecular corrugations are still visible, but more disordered compared to pristine SAM. The desorption of single molecular moieties cannot be excluded. (e) Size distribution of the observed dark spots (in black) as a function of the molecular area (0.288 nm^2^) in the β-phase. Simulated size distributions of cross-linked molecules with an area fraction of 3% and 30%, respectively, based on step-growth kinetics.

Investigating the initial stages of cross-linking allows for the visualization of the effects of electron impact at the local molecular level. The pristine TPT SAM prepared from the gas phase was shown in Figure 3a. The irradiation of SAMs with either 1 keV (Figure 3b, 0.5 mC/cm^2^) or with 50 eV (Figure S3 in Supporting Information File 1) electrons induces the formation of “dark spots” of various sizes within the domains. The dark spots are apparently surrounded by the pristine monolayer. Features similar to dark spots with a diameter of ≈10 Å were observed by Kondoh et al. on the methylthiolate/Au(111) surface after electron irradiation at 4 keV and assigned as sulfur species after cleavage of S–C bonds at the interface [69]. In our case, a higher magnification view reveals that molecular corrugation features are still visible inside the spots (Figure 3d). Therefore, the dark spots observed in TPT monolayers are not merely sulfur species due to electron-induced desorption of molecules since they still contain molecules and/or molecular fragments. However, we cannot completely rule out the possibility of single-molecule desorption. The apparent depth of these dark spots is 1.4 ± 0.1 Å and the spatial distribution can be approximated by a Poisson distribution (Figure S5 in Supporting Information File 1), implying that these dark spots are random and independent of each other. In addition, their size distribution was plotted as a function of the area (0.288 nm^2^) occupied by individual molecules in the β-phase (Figure 3e). It is evident that the size of most spots lies within 1–5 molecules, but large spots can contain up to 43 molecules. The areal number density of the dark spots is estimated to be (2.0 ± 1.0) × 10^12^ cm^−2^ and the corresponding area fraction is approximately 3.2%. Analogous to the formation of 2D polymers on a surface or interface [6], the cross-linking could proceed either by step-growth or chain-growth kinetics. In the step-growth scenario, the polymerization is proceeded by random dimerization of the neighboring monomers and a local dimerization event neither promotes nor inhibits dimerization of the neighboring sites. We performed a simple simulation assuming that one molecule can form covalent bonds with six neighboring molecules via random dimerization (Figure S6 in Supporting Information File 1). The size distribution of reacted molecules within a cell of 29700 nm^2^ is plotted in comparison to that of the dark spots determined from STM (Figure 3e). The cross-linked areas, for the same area fraction of 3% of the STM data, consist of almost exclusively dimers and, to a much lesser extent, trimers. An area fraction of 30% results in areas with up to ten monomers. The comparison reveals inconsistencies between the STM data and the step-growth kinetics. Therefore, we look into the chain-growth scenario, in which a radical initiates a reaction with an adjacent molecule and generates another new radical carbon center, which may react with other molecules. According to Amiaud et al. [53], the formation of first radicals can be caused either by electronic rearrangement or dissociative electron attachment of the negative ion resonance. For the irradiation dose of 0.5 mC/cm^2^ with 1 keV primary electrons, an areal number density of (1.7 ± 0.7) × 10^13^ cm^−2^ was obtained for secondary electrons with kinetic energies within the window of the resonance (see detailed calculations in Supporting Information File 1). When considering the propagation of radical chain reactions with an average of 5–6 monomers, we estimated an areal number density of (3.8 ± 1.9) × 10^12^ cm^−2^, which is in good agreement with the areal number density of dark spots in the STM image. Since cross-linking occurs in two dimensions in order to form a mechanically stable sheet, a molecule located at a node must be able to connect with at least three adjacent molecules. Furthermore, because these molecules are immobilized on the surface, the constraints of the spatial geometry greatly reduce the possibility of radical chain elongation, which explains why the larger dark spot region in the initial cross-linking stage is limited to a maximum of 43 molecules.

### Reduction of the structural order and observation of subnanometer voids

**Figure 4:**
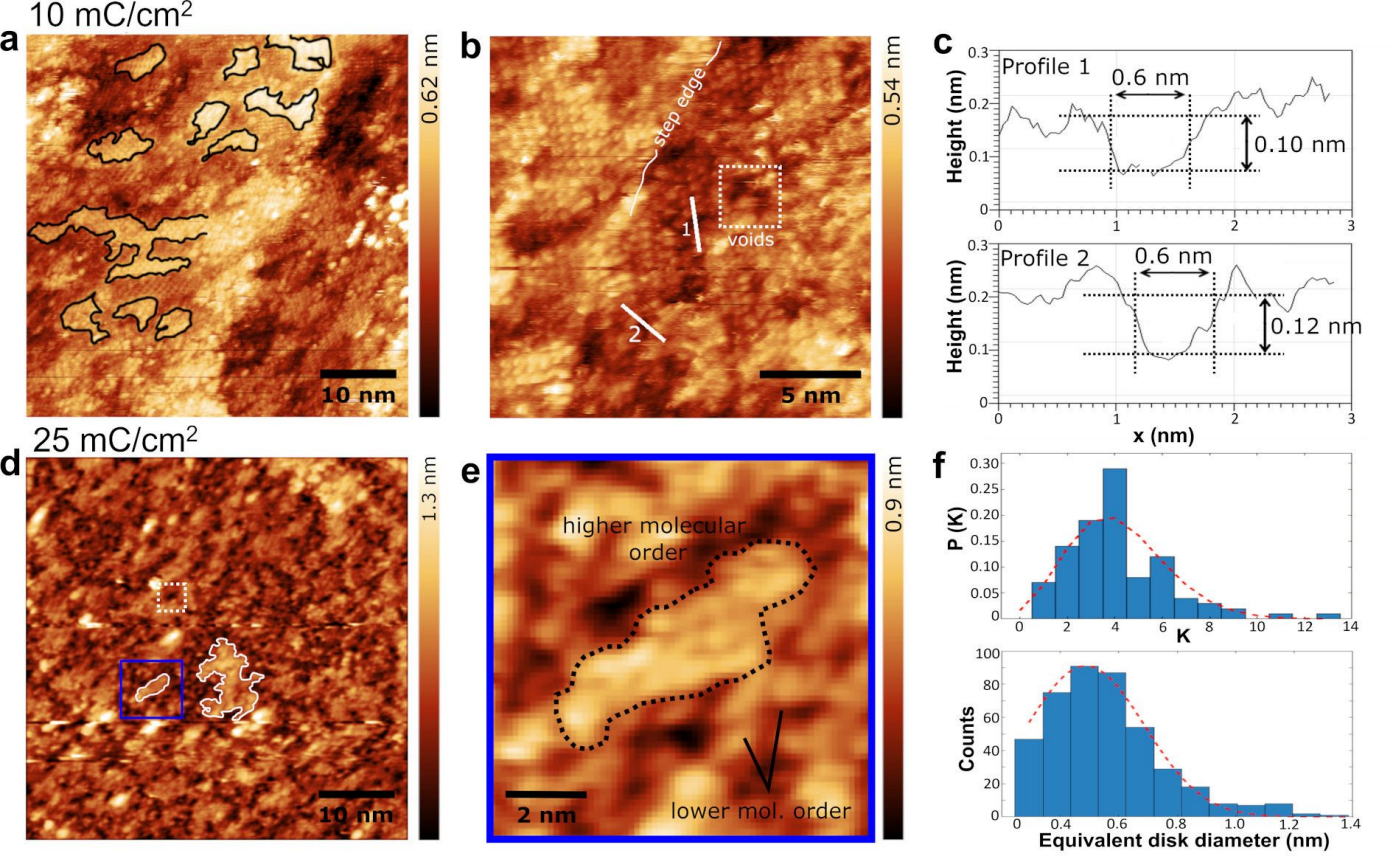
Structural evolution of TPT SAMs upon 50 eV electron exposure. (a) Low-magnification STM image (–1.2 V, 10 pA) shows that electron exposure (10 mC/cm^2^) causes a reduction in the long-range order. Regular phase domains are ≈10 nm and are surrounded by cross-linked regions. (b) High-magnification STM image of Figure 4a shows the formation of subnanometer-sized voids (highlighted by the white square). (c) Height profiles along lines 1 and 2 marked in Figure 4b. The measured depth is ≈0.1 nm. (d) High-magnification STM image of the monolayer irradiated at a dose of 25 mC/cm^2^. The voids are preferentially located in darker regions of the monolayer. This image was generated by post-processing of Supporting Information File 1, Figure S8a using the continuous wavelet transform function. (e) A high magnification view of the marked area in Figure 4d highlights the remainders of the ordered layer. (f) Top panel: lateral distribution of the voids. The fit (red dashed curve) follows a Poisson distribution with parameter λ = 3.7. Bottom panel: estimation of void diameter by calculating the equivalent disk diameter, which is the diameter of circular voids of the same area.

TPT SAMs prepared from a DMF-based solution were irradiated with 50 eV electrons at 2.5, 10, 15, or 25 mC/cm^2^. The SAM irradiated with 2.5 mC/cm^2^ is characterized by dark spots (Figure S7b in Supporting Information File 1), attributed to locally cross-linked molecules. Increasing the electron exposure to 10 mC/cm^2^ leads to a significant reduction of the long-range order (Figure 4a), whereby the STM images still show phase domains (marked in black) with sizes smaller than 10 nm. These slightly affected domains seem to be “embedded” in a rather amorphous matrix, which appears darker compared to the ordered domains and cannot be molecularly resolved by STM. At a dose of 10 mC/cm^2^, we observed the occurrence of subnanometer-sized voids within the less ordered molecular constituents (highlighted by a white square in Figure 4b). Since the apparent height of these voids is ≈0.1 nm lower than that of the cross-linked molecules (Figure 4c), we use the term “voids” to distinguish them from the aforementioned dark spots. Note that the molecular layer still exhibits structural order in the form of small regular domains, typically smaller than 10 nm in size.

Upon electron irradiation with a dose of 15 mC/cm^2^, TPT SAMs were subject to a progressive loss of structural order (Figure S7c, Supporting Information File 1). A further exposure at a dose of 25 mC/cm^2^ leads to an almost complete loss of the long- and short-range molecular order. The original STM image (Figure S8a, Supporting Information File 1) was post-processed by a continuous wavelet transform function and a two-pixel-scaled Gaussian wavelet type [70], as shown in Figure 4d. Brighter islands of 5–10 nm in size were still visible, separated by darker structures with a branched form, which can be considered remainders of the ordered layer. Many voids can be observed here. Unlike the dynamic free volume pores of polymers, these voids remain stable after multiple STM scans. By counting the number of voids in a region, an areal density of ≈1.7 × 10^17^ voids/m^2^ (Figure S8b, Supporting Information File 1) is calculated. The lateral distribution of these voids reveals a small deviation from the Poisson distribution (Figure 4f, top panel), as more voids are found in the darker regions and almost no voids are found in the brighter regions. This is consistent with Figure 4a, where regions with remaining molecular order appear brighter than cross-linked regions. The equivalent size distribution of the voids shows an average diameter of 0.5 ± 0.2 nm (Figure 4f, bottom panel). These values determined by STM are smaller than the areal density of ≈7 × 10^17^ m^−2^ and the pore diameter of 0.7 ± 0.1 nm determined using AFM [36]. This is mainly due to different imaging mechanisms and different threshold definitions used for pore determination.

**Figure 5:**
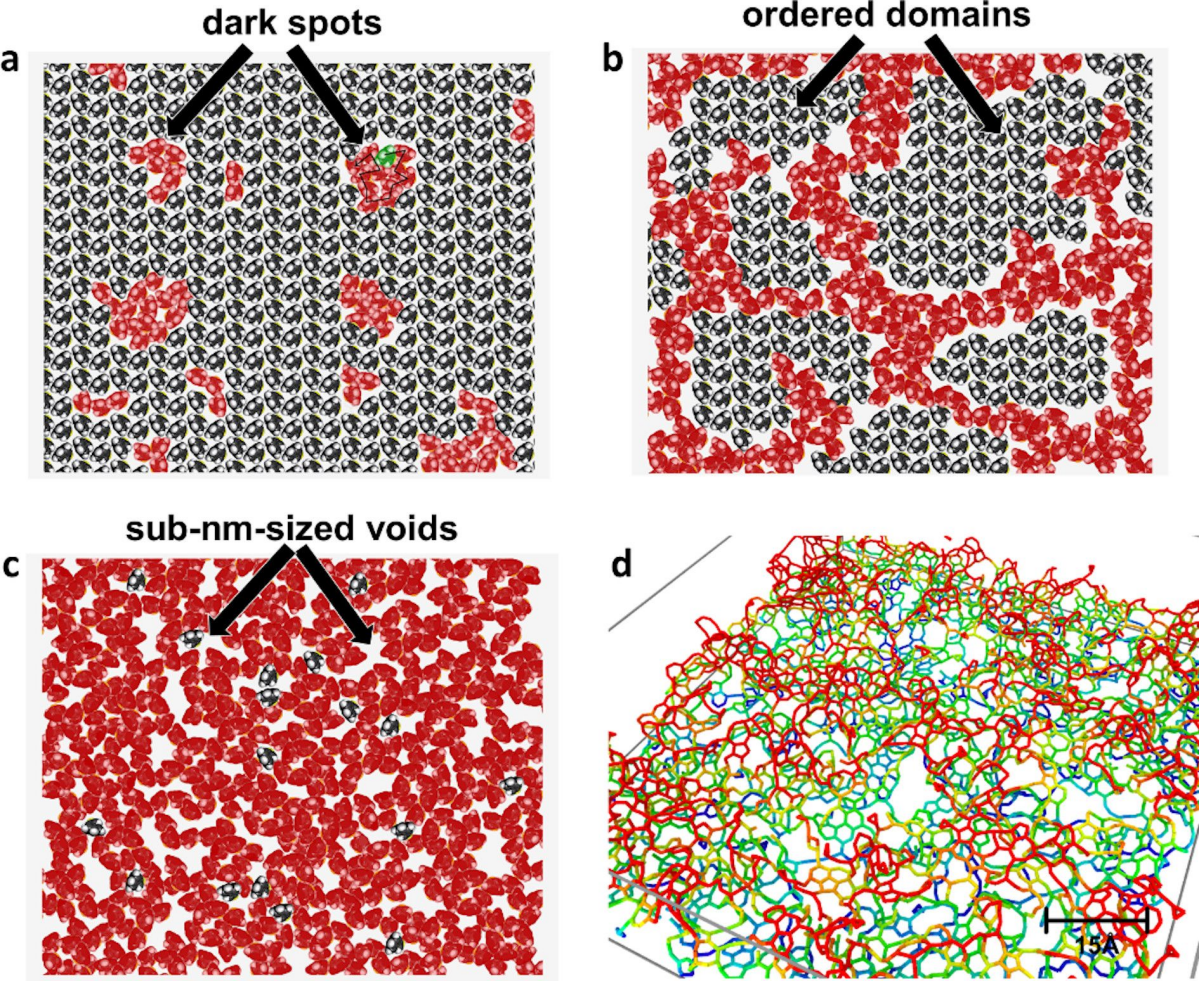
Schematic visualization of the proceeding of cross-linking via radical chain reactions under two-dimensional configurational constraints. (a) At low doses, cross-linked molecules appear as dark spots in the STM image. (b) At intermediate doses, the cross-linked branches spread in random directions and the size of pristine domains continues to decrease. (c) At high doses, the SAM is transformed into an amorphous network of carbon atoms with subnanometer-sized voids. (d) Example wireframe structure of a TPT-based CNM calculated via LAMMPS using the EDIP potential: the colors denote height (blue to red). Several voids are visible here. The simulation box contains 20 × 20 × 18 carbon atoms. Figure 5d was adapted with permission from [71] (J. Ehrens; F. Gayk; P. Vorndamme; T. Heitmann; N. Biere; D. Anselmetti; X. Zhang; A. Gölzhäuser; J. Schnack, Phys. Rev. B, vol. 103, article no. 115416, 2021). Copyright (2021) by the American Physical Society. This content is not subject to CC BY 4.0.

Finally, the evolution of electron-induced cross-linking of the TPT SAM observed by STM is schematically illustrated as follows (Figure 5a–c). At low doses, cross-linked domains appear as dark spots in the STM images. At the medium dose, the cross-linking causes variation in the local density and the structural order of the monolayer. Again, the cross-linked regions appear darker than the structurally ordered domains. At high doses, subnanometer-sized voids are formed preferably in regions of lower structural order and lower density in the monolayer. The formation of carbon nanomembranes was also modeled using classical molecular dynamics simulations. Figure 5d shows a typical structure of a TPT CNM, where pronounced valleys and hills in both height and lateral distance from each other can be clearly seen. In a simulation box containing 20 × 20 × 18 carbon atoms, the TPT CNM exhibits three subnanometer pores, corresponding to an areal density of ≈3.3 × 10^16^ voids/m^2^. A detailed theoretical study on the formation of CNMs has been separately published [71].

## Conclusion

The STM investigation provided an intuitive recognition of electron-induced structural changes when *p*-terphenylthiol SAMs on Au(111) are exposed to 50 eV and 1 keV electrons. Dark spots appeared in the initial stage of cross-linking. The cross-linked regions speedily grow and form an amorphous carbon matrix with reduced structural order. The fully cross-linked monolayer is characterized by the formation of subnanometer-sized voids preferably in regions of lower structural order and lower density, which enables the use of CNMs as a molecular sieve in mass transfer and separation processes.

## Experimental

### Preparation of self-assembled monolayers from solution

Precursor molecules (*p*-terphenylthiol) used in this study were purchased from Sigma-Aldrich. In a manner similar to the reference [34], we used a 300 nm thermally evaporated Au(111) layer on mica substrates (Georg Albert PVD-Coatings, Germany). These substrates provide, without further flame-annealing treatment, atomically flat terraces in the range of 100 nm (terraces of 100–200 nm were typically observed). The substrate was cleaned in a UV/ozone cleaner (UVOH 150 LAB FHR) for 3 min, rinsed with ethanol, and then blown dry under a nitrogen stream. The substrates were immersed into a ≈1 mM solution of TPT in dry and degassed dimethylformamide. The solution was then heated at 70 °C in a sealed flask under a nitrogen atmosphere for 24 h. Afterward, the sample was taken out from the solution, rinsed with DMF, and EtOH and blown dry with nitrogen.

### Preparation of self-assembled monolayers from the gas phase

The TPT molecules were deposited from the gas phase on Au(111)/mica substrates under UHV conditions. Firstly, a clean Au(111) surface was prepared by argon sputtering for 10 min at 1 keV with a pressure of 3 × 10^−6^ mbar. Secondly, the treated substrate was annealed at 673 K for 1 h in order to obtain a flat substrate surface characterized by large gold terraces. When required, successive sputtering/annealing cycles were performed. Immediately after sputtering, the gold substrate was exposed to the molecular beam from a quartz crucible inside a Knudsen-type organic evaporator (TCE-BSC, Kentax). The crucible was filled with TPT crystals previously purified by sublimation. The sublimation temperature was set to 398 K and the substrate was held at room temperature. The chamber pressure during the evaporation process was ≈10^−8^ mbar and the evaporation time was ≈30 min. X-ray photoelectron spectroscopy was employed to monitor the existence of thiolates as well as physisorbed thiols. To remove physisorbed molecules, the sample was further annealed at 343 K for ≈15 min.

### Irradiation of TPT SAMs

The TPT SAMs were irradiated under UHV conditions with electrons from a floodgun (FG20, Specs, Germany) with an energy of 50 eV. The irradiation dose was calibrated by using a Faraday cup. The TPT SAMs were irradiated by the rastering electron beam of an SEM, where the kinetic energy of the electrons was set to 1 keV. The SEM used is a modified type of a UHV Zeiss Standard Gemini with a Schottky-type thermal field emission source (ZrO/W). The pressure in the SEM column was ≈10^−8^ mbar. Prior to each experiment, the beam current was measured by using a Faraday cup. The SAMs were irradiated with beam currents ranging from 200–600 pA. Prior to electron irradiation, the STM tip was retracted and moved away from the surface location of interest, such that the electrons could pass the tip and reach the surface location. The irradiated area was typically around 30 × 40 µm^2^. The time per cycle was 2.5 s.

### Imaging of SAMs by STM

The STM experiments were performed on a commercial Omicron Multiscan system combining both a temperature-variable STM (Multiscan STM VT) and an SEM. The STM tip is aligned at ≈45° with respect to the surface normal, thus enabling to control the position of the STM tip by SEM. All STM data was acquired at room temperature under UHV conditions (chamber pressure <10^−10^ mbar). The STM was operated in constant-current mode with tunneling currents of 10–100 pA and sample biases between −1.2 to −0.4 V and +0.4 to +1.2 V. A *z*-resolution higher than 0.01 nm could be achieved. The STM tips were prepared from 0.375 mm polycrystalline tungsten wire (Alfa Aesar) by electrochemical etching in a 3 M NaOH solution. The instrument was calibrated by imaging HOPG with atomic resolution. The data was post-processed by using the Gwyddion v.2.41 free software.

### Analysis of dark spots and subnanometer voids in STM images

The dark spots and subnanometer voids in STM images were marked by employing the segmentation function of Gwyddion 2.41. Only the spots inside the phase domains were marked, whereas the domain boundaries were omitted. The size distribution of the dark spots was plotted as a function of the spot area, which is represented in units of 0.288 nm^2^, corresponding to the molecular area in the β-phase. The spatial distribution of the dark spots was evaluated by dividing the STM image into equal sections and then the number of spots was counted in each section. The mean depth distribution of the dark spots was obtained by determining the mean depth for every dark spot with respect to the SAM/ambient interfacial area. This was done by averaging the measured depth for each individual pixel. The data were analyzed by using an empirical fit. The lateral distribution of subnanometer voids was analyzed by partitioning the surface area into small segments and counting the number of voids in each segment.

### Classical molecular dynamics simulations

The formation of a CNM was modelled using classical molecular dynamics. The LAMMPS package was employed and the EDIP as well as AIREBO carbon potentials were used depending on more technical simulation-specific parameters. As reported in the reference [71], the simulations included only carbon atoms. The modelling was achieved through the following steps: (1) The formation of a TPT SAM on Au(111) was initiated by placing carbon atoms above a surface at positions they would have in the STM image, where the underlying gold substrate was replaced by a repulsive Lennard-Jones wall potential (initialization). (2) Specific starting conditions were imposed by tilting or randomly moving some or all molecules and by either removing some of the atoms or whole molecules to, for example, mimic defects in the experimental process (randomization). (3) The electron irradiation was modeled by a vertical force gradient being applied to the atoms; it is linear and decreases with height (compression). The effect of secondary electrons was modeled by lateral forces on randomly selected molecules. (4) The model system was then allowed to relax toward an equilibrium structure according to thermostat dynamics (Nosé–Hoover or Langevin) with a temperature that linearly decreases with time (cooling). This corresponds to the fact that the gold support also acts as a very efficient heat sink during the synthesis process. Figure 5d is a typical result of vertical and lateral momentum dynamics applied to TPT SAMs after 5700 time steps with following parameters: *T* = 700 K, *v* = 35 Å/ps, *k* = 30 eV/Å.

## Supporting Information

File 1 Additional figures and detailed calculations.
