# Supplementary material for: Investigation of electron-induced cross-linking of self-assembled monolayers by scanning tunneling microscopy
Source: Beilstein J Nanotechnol. 2022 May 25;13:462–71. doi: 10.3762/bjnano.13.39 (PMC9152271; doi:10.3762/bjnano.13.39)
Supplement: File 1 — Additional figures and detailed calculations. [file Beilstein_J_Nanotechnol-13-462-s001.pdf]

BEILSTEIN JOURNAL OF NANOTECHNOLOGY

Supporting Information

for

Investigation of electron-induced cross-linking of self-assembled monolayers by scanning tunneling microscopy

Patrick Stohmann, Sascha Koch, Yang Yang, Christopher David Kaiser, Julian Ehrens, Jürgen Schnack, Niklas Biere, Dario Anselmetti, Armin Gölzhäuser and Xianghui Zhang

Beilstein J. Nanotechnol. **2022,** *13,* 462–471. doi:10.3762/bjnano.13.39

Additional figures and detailed calculations

License and Terms: This is a supporting information file under the terms of the Creative Commons Attribution License (https://creativecommons.org/licenses/by/4.0). Please note that the reuse, redistribution and reproduction in particular requires that the author(s) and source are credited and that individual graphics may be subject to special legal provisions.
The license is subject to the *Beilstein Journal of Nanotechnology* terms and conditions: (https://www.beilstein-journals.org/bjnano/terms)

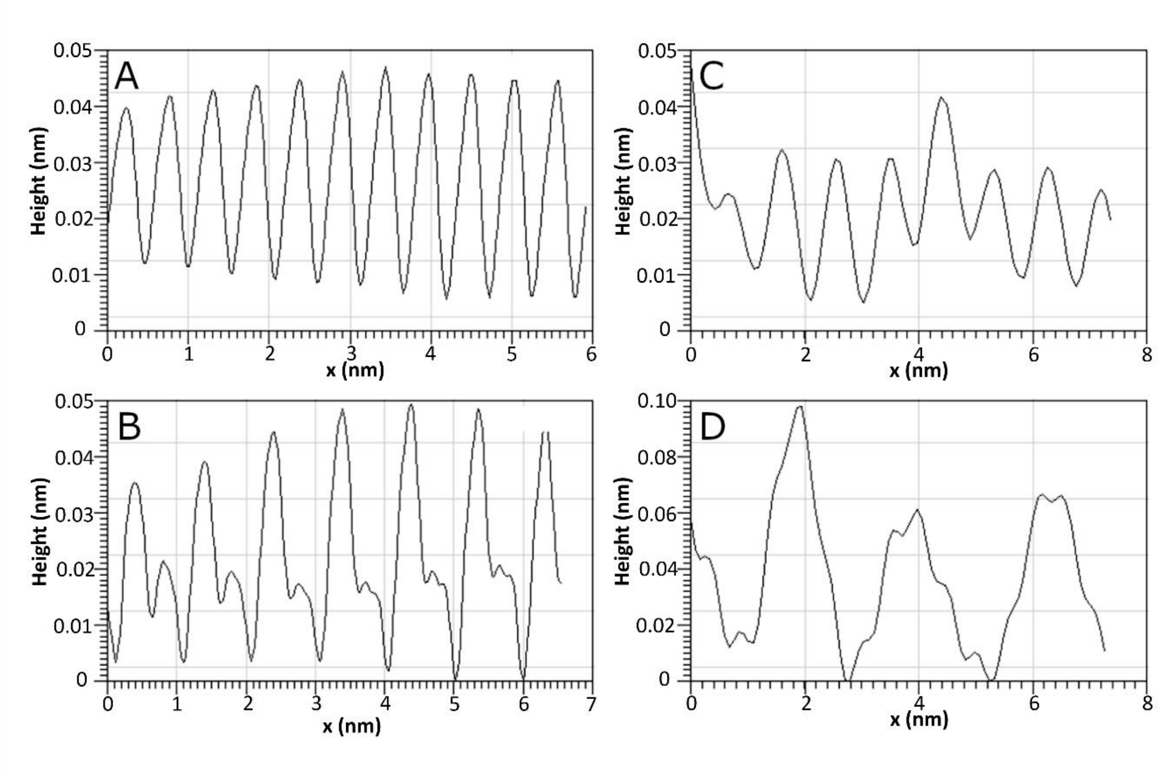

Figure S1: Height profiles of lines A–D of Figure 2b in the main manuscript.

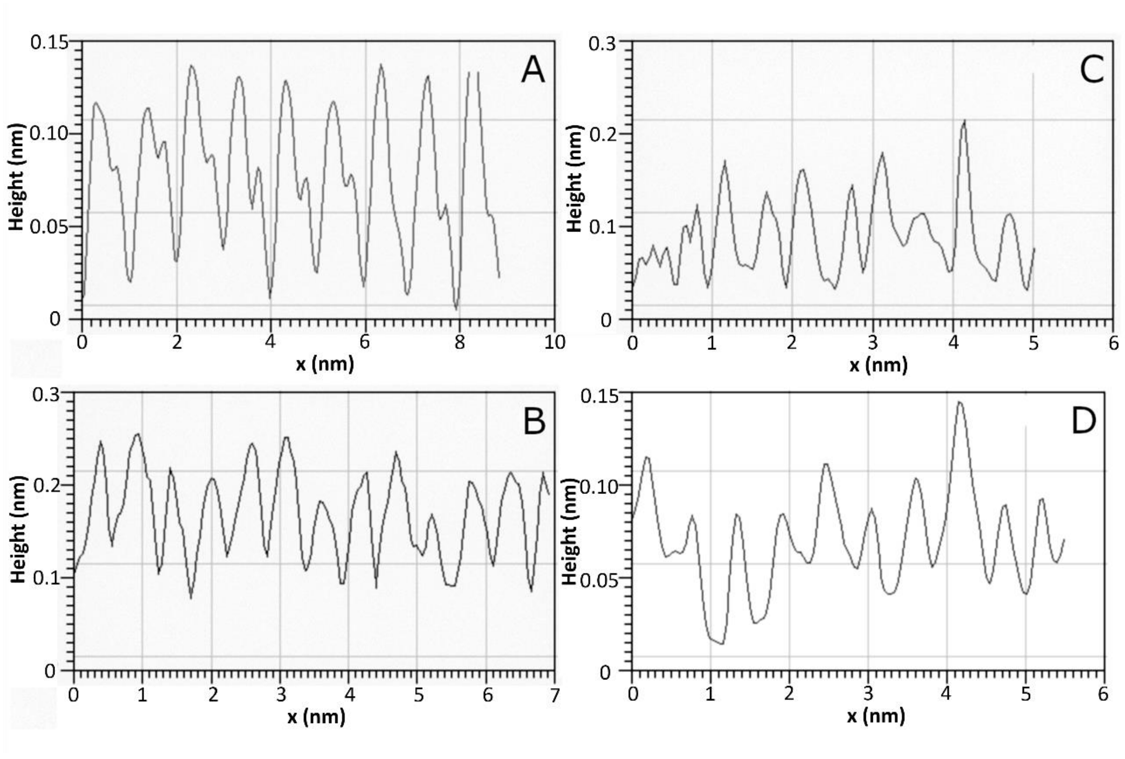

Figure S2: Height profiles of lines A–D of Figure 2d in the main manuscript.

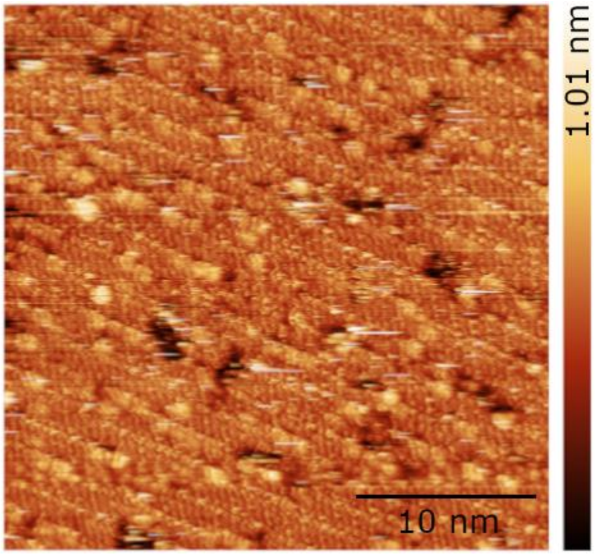

Figure S3: A TPT SAM irradiated with 50 eV electrons at the same electron dose of 0.5 mC/cm^2. Dark spots are also visible here.

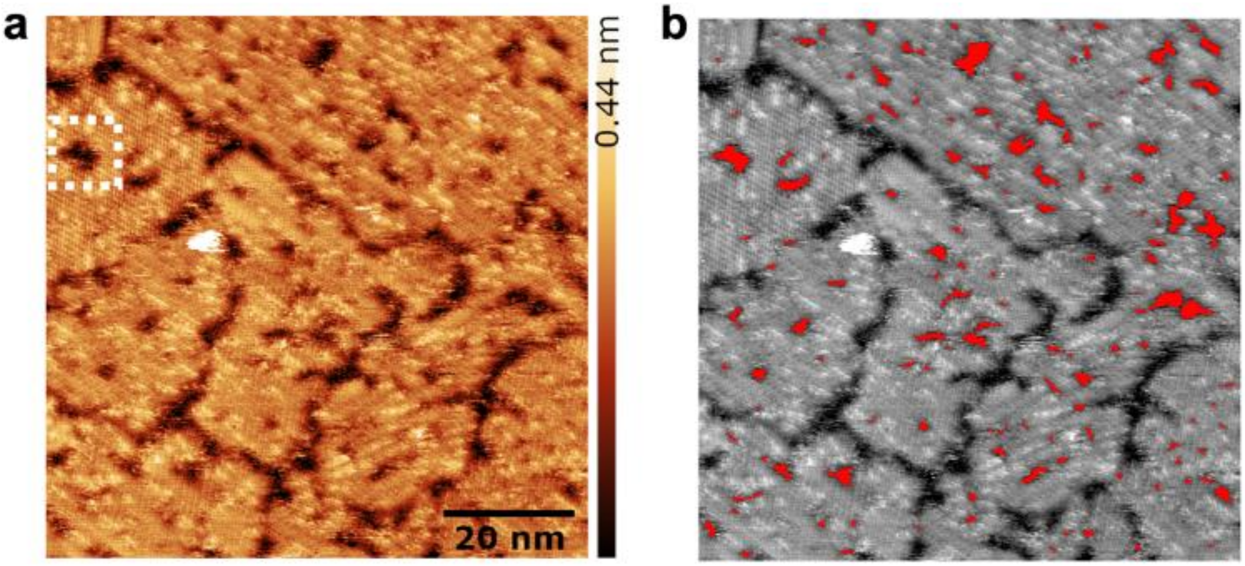

Figure S4: Quantitative analysis of the dark spots. (a) STM image (0.45 V, 70 pA) of the monolayer irradiated with 1 keV electrons at a dose of 0.5 mC/cm^2. (b) Dark spots are marked in red by the segmentation function of Gwyddion 2.41.

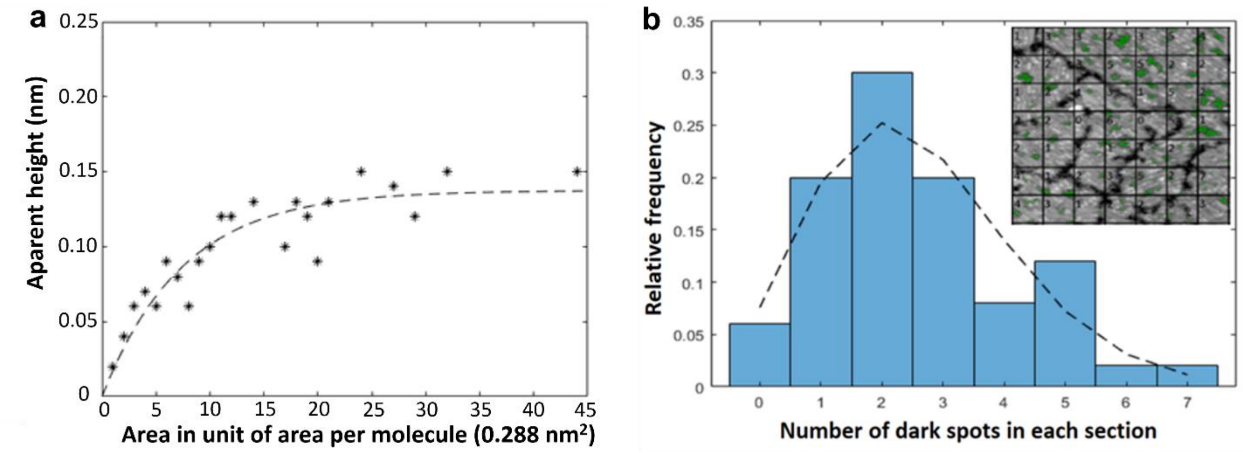

Figure S5: (a) The apparent depth of dark spots as a function of area in units of area per molecule. (b) The spatial distribution of the dark spots was evaluated by dividing the STM image into equal sections and then counting the number of spots in each section. The spatial distribution can be approximated by a Poisson distribution, indicating that the dark spots are random and independent of each other.

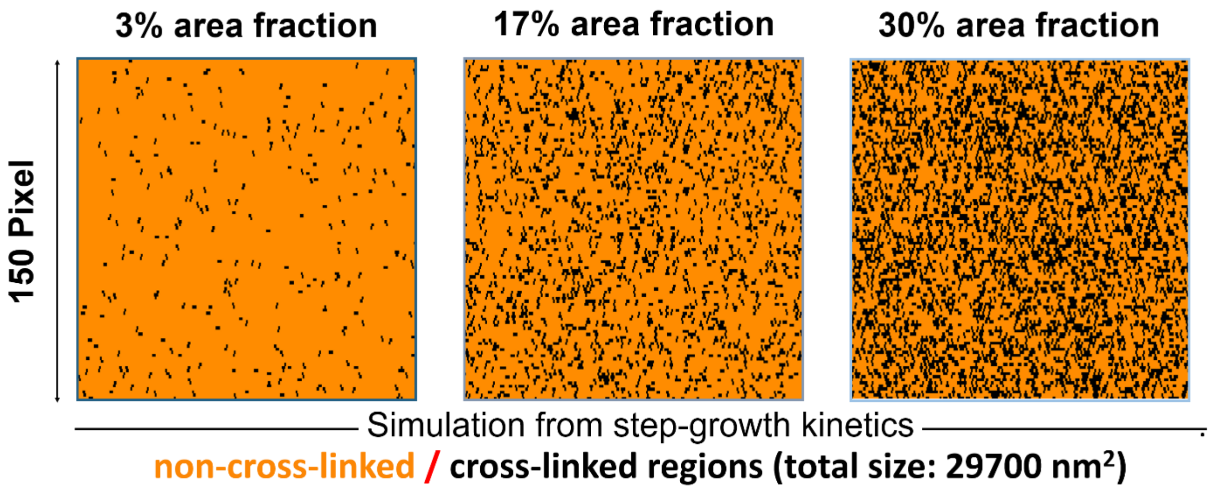

Figure S6: Simulated cross-linked (in black) and non-cross-linked (in orange) molecules according to the step-growth model. The area fractions are 3%, 17%, and 30%, respectively. The size distribution of reacted or cross-linked molecules obtained by simulation are plotted in comparison to the size distribution of the dark spots (also see Figure 3h in the main manuscript) observed by STM. The area in STM imaging is 29700 nm^2 and the simulated data is multiplied by a factor of 29700/6400. One pixel represents one molecule.

Expected areal number density of dark spots in the chain-growth scenario [1]

According to Amiaud et al. [2], cross-linking within a TPT SAM may proceed via radical chain reactions starting with a resonant electron attachment process at 6 eV. The formation of the first radicals initiating chain reactions each may then proceed via electronic rearrangement or via dissociative electron attachment (DEA). As the irradiation was performed with 6 eV primary electrons, that is, below ionization and excitation thresholds, the other (non-resonant) scattering processes, which lead to the formation of the first radicalized monomers such as neutral dissociation (ND) or dissociative ionization (DI), could be excluded. TPT on Au(111) may have the first ionization potential between 6 and 9 eV. However, as our irradiation experiments were performed with 50 eV and 1 keV primary electrons, all electron-induced fragmentation pathways need to be taken into account in the first place.

It is assumed in the following that the dark spots observed in the STM images are formed upon generation of one (first) radical each of which subsequently initiates radical chain reactions. To investigate the potential contribution of the emitted 6 eV secondary electrons (SE) to the creation of the first radicals, the areal number density of the dark spots observed in the STM image shown in Figure S4b, n_{spots}^{STM}, is compared with the expected areal number density of reactive electron attachment (EA) events, n_{rEA}. Reactive EA events are defined as EA events that eventually lead to the formation of at least one intermolecular carbon–carbon bond. n_{rEA} can be estimated by employing the (over)estimated reactive EA cross-section $\sigma_{rEA}^{HREELS} \sim 1.2 \times 10^{-16} \mathrm{cm}^2$ introduced by Amiaud *et al.* based on the HREELS data [2], in particular on the observed loss of aromaticity.

The expected areal number density of reactive EA events is estimated by employing the following equation:

$$n_{rEA} = n_{SE,6eV} \times \frac{\sigma_{rEA}^{HREELS}}{A_{mol}}$$

Here, A_{mol} denotes the area occupied by a single molecule in the β-phase, which is 0.288 nm^2, and $n_{SE,6eV}$ denotes the areal number density of secondary electrons (SE) emitted within the window of the resonance, that is, with kinetic energies of 6.0 ± 1.5 eV. $n_{SE,6eV}$ can be determined by employing the following equation:

$$n_{SE,6eV} = SEY \times f_{SE,6eV} \times n_{PE}$$

where SEY denotes the PE kinetic energy-dependent secondary electron yield, $f_{SE,6eV}$ denotes the fraction of the overall distribution of SE produced within the window of the resonance, and d_{PE} denotes the PE areal number density.

The *SEY* for incident 1 keV PE impinging on gold surfaces was determined by Gonzales et al., yielding 1.65 (clean surface) and 1.85 (contaminated surface) [3]. $f_{SE,6eV}$ was graphically estimated to be 5–10% by Houplin et al. for 50 eV PE impact [4]. This value should be similar in the case of 1 keV PE electron impact as the low-energy tail of the SE energy distribution does not significantly change with the increase in the PE kinetic energy. n_{PE} is derived by dividing the applied PE dose d_{PE} by the elementary charge *e*, yielding n_{PE} = 0.5 ± 0.1 mC · cm^{-2}/e = 3.1 ± 0.7 × 10^{15} cm^{-2}. Accordingly, $n_{SE,6eV}$ is estimated to be 4.1 ± 1.7 × 10^{14} cm^{-2}.

Therefore, the expected areal number density of reactive EA events, upon 1 keV electron exposure with a dose of 0.5 mC/cm^2, amounts to n_{rEA} = 1.7 ± 0.7 × 10^{13} cm^{-2}. This value is to be compared to the areal number density of the dark spots observed in the STM image shown in Figure S4b, which amounts to n_{spots}^{STM} = 2.0 ± 1.0 × 10^{12} cm^{-2}. By contrasting the areal number densities it is seen that n_{spots}^{STM} is lower than n_{rEA} by roughly one order of magnitude. This result appears counterintuitive as n_{rEA} was derived from the reactive EA cross-section determined on the basis of HREELS data [2]. Assuming that each dark spot is created upon the formation of a TPT radical monomer, n_{spots}^{STM} is expected

to be equal to or higher than n_{rEA}, in particular, as further reaction pathways (ND and/or DI) may contribute to the formation of radical monomers at higher energies [5].

However, the reactive EA cross-section σ_{rEA}^{HREELS} was overestimated by Amiaud et al. HREELS data reveals that a 6 eV electron irradiation with a dose of 50 electrons per molecule leads to a decrease of 47–53% of the aromatic CH stretching feature. As one TPT monomer has 13 aromatic CH groups, 6–7 aromatic carbon centers, on average, are converted into aliphatic carbon centers after irradiation. Without considering the propagation of radical chain reactions, that is, only the reaction between two monomers is taken into account, the creation of one radical center causes the formation of two aliphatic groups. Provided that every DEA event leads to a reaction with an adjacent monomer, three DEA events per monomer are required, on average, to cause the observed ≈50% loss of aromaticity. Considering that every monomer is irradiated by 50 electrons and occupies an area of ≈20 Å^2, the reactive EA cross-section is $\sigma_{rEA}^{HREELS} \sim 1.2 \times 10^{-16} \mathrm{cm}^2$.

Amiaud et al. overestimated the reactive EA cross-section by neglecting the propagation of radical chain reactions. Therefore, the theoretical considerations of Amiaud et al. are extended in the following: When considering the propagation with *n* monomers involved on average, every DEA event should cause the formation of $2n - 2$ aliphatic groups within the monolayer, which means that $n-1$ times more aliphatic groups are created compared to the case when the propagation is neglected. Therefore, the (over)estimated reactive *EA* cross-section $\sigma_{rEA}^{HREELS} \sim 1.2 \times 10^{-16}$ cm^2 is to be divided by ($n - 1$). The STM data indicates that, on average, 5–6 monomers are involved in the radical chain reactions. Considering the propagation of radical chain reactions with $n = 5 – 6$ monomers involved, the reactive EA cross-section is reduced to $\sigma_{rEA}^{HREELS} \approx 1.2 \times 10^{-16}$ cm^2/($n - 1$) ≈ 2.2 ± 0.3 × 10^{-17} cm^2. Therefore, this allows for the estimation of the expected areal number density of reactive EA events upon 1 keV electron exposure with a dose of 0.5 mC/cm^2, which is n_{rEA} = 3.8 ± 1.9 × 10^{12} cm^{-2}. When contrasting this value with n_{spots}^{STM} = 2.0 ± 1.0 × 10^{12} cm^{-2}, the areal number density of the dark spots observed in the STM image shown in Figure S4b is in good agreement with the expected areal number density of reactive *EA* events when considering radical chain reactions.

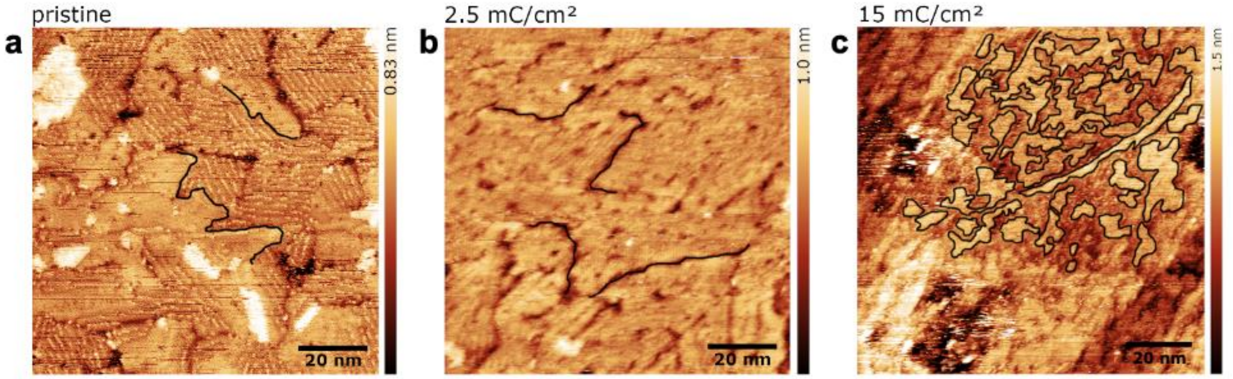

Figure S7: (a) Pristine TPT SAMs prepared from solution. Structural evolution of the TPT SAMs upon 50 eV electron exposure at doses of (b) 2.5 mC/cm^2, (c) 15 mC/cm^2.

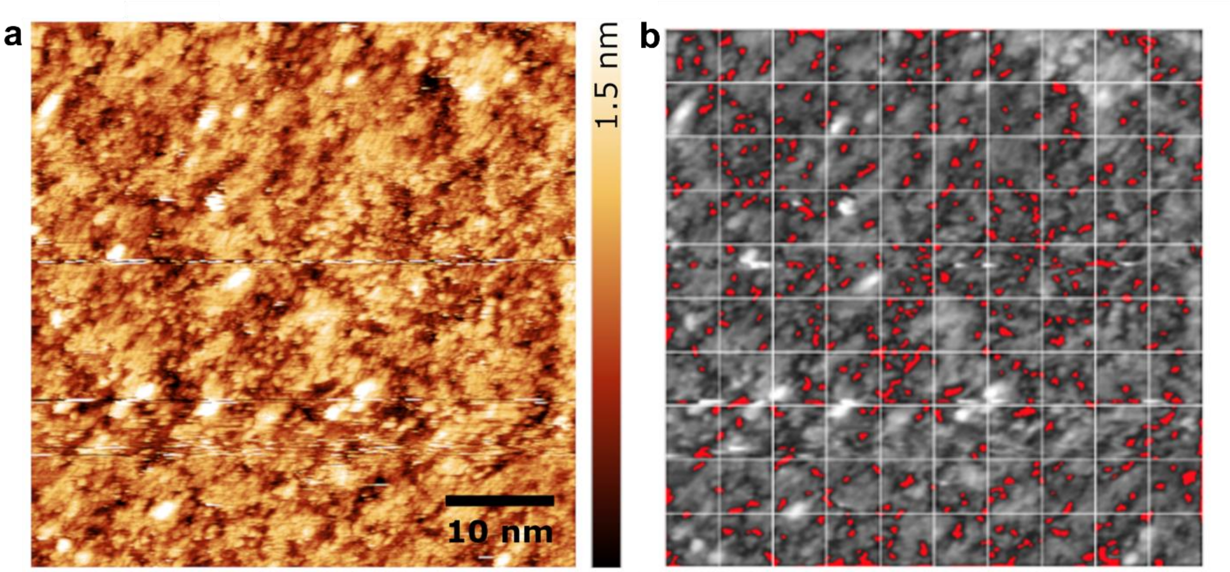

Figure S8: Quantitative analysis of subnanometer voids. (a) STM image of a TPT monolayer irradiated by 50 eV electrons at a dose of 25 mC/cm^2. This image was post-processed using the continuous wavelet transform-function and is shown in Figure 4d. (b) Subnanometer voids are marked in red by using the segmentation function of Gwyddion 2.41.

References

1. Stohmann, P. *Investigation of Electron Irradiation-Induced Cross-Linking of p-Terphenyl Thiol Self-Assembled Monolayers on Au(111) by Scanning Tunneling Microscopy, PhD Thesis 2020, Bielefeld University, Germany.*
2. Amiaud, L.; Houplin, J.; Bourdier, M.; Humblot, V.; Azria, R.; Pradier, C.-M.; Lafosse, A. *Phys. Chem. Chem. Phys.* **2014,** *16*, 1050–1059. doi:10.1039/c3cp53023j
3. Gonzalez, L. A.; Angelucci, M.; Larciprete, R.; Cimino, R. *AIP Advances* **2017,** *7*, 115203. doi:10.1063/1.5000118
4. Houplin, J.; Dablemont, C.; Sala, L.; Lafosse, A.; Amiaud, L. *Langmuir* **2015,** *31*, 13528–13534. doi:10.1021/acs.langmuir.5b02109
5. Böhler, E.; Warneke, J.; Swiderek, P. *Chemical Society reviews* **2013,** *42*, 9219–9231. doi:10.1039/C3CS60180C
